# The American International Congress on Tuberculosis

**Published:** 1906-07

**Authors:** 


### THE AMERICAN INTERNATIONAL CONGRESS ON TUBERCULOSIS. NEW YORK, NOV. 14, 15, 16.

The following correspondence is published for the information of the profession and of all persons interested in this great humanitarian work. The Congress is thus under the ægis of the Federal government, has its endorsement, support and the seal of its authority. If there has been any doubt on the success of the Congress this high approval will remove it.

DEPARTMENT OF STATE.

WASHINGTON, June 5, 1906.

*Clark Bell, Esquire, Treasurer of the American International Congress on Tuberculosis, 39 Broadway, New York.*

SIR: In reply to your letter of April 2d last, I have to inform you that on May 31 instructions were sent to American diplomatic officers in American States to support the invitation extended by the American International Congress on Tuberculosis to send delegates to its meeting in November next.

In compliance with your request, the instructions sent by Mr. Hay concerning the meeting of the Congress at St. Louis was embodied in the instructions sent on the 31st ultimo. I enclose a printed copy of them.

On the same day the American diplomatic representatives in Great Britain, France, Denmark and the Netherlands were directed to support the invitation to their respective American colonial possessions to be represented at the Congress.

I am, sir, your obedient servant,

ROBERT BACON,

Acting Secretary.

Enclosure: To American diplomatic officers in American States, May 31, 1906.

### AMERICAN INTERNATIONAL CONGRESS ON TUBERCULOSIS.

DEPARTMENT OF STATE.

WASHINGTON, May 31, 1906.

*To the Diplomatic Officers of the United States in American States.*

GENTLEMEN: The Department is informed by the Executive Committee of the American International Congress on Tuberculosis that they have sent to the Government of each American

Country an invitation for official representation by that government in the next session of the Congress, which will be held at the city of New York during the three days of November 14, 15 and 16, 1906; and the request is made of the Department to give such support to the invitation as it properly may.

In instructing the diplomatic officers to give their support to a similar invitation extended by the Congress for their St. Louis meeting in 1904, my predecessor, Mr. Hay, said:

"The humanitarian object which this Congress has in view—to reach by the discussion of scientific men some result in arresting the spread and averting, so far as it may be found possible, the ravages of this dreadful disease, which now falls with such terrible force and fatality upon the people of the Western Hemisphere—can not but enlist the sympathy and approval of the government to which you are accredited.

"The Department will, therefore, be pleased to have you say to that government that this government is in entire sympathy with the work of the proposed Congress, and would be pleased to learn that the Government of........took a like interest in its success by the acceptance of the committee's invitation and the appointment of three or more scientific gentlemen to represent it at the Congress.

"This government would also be pleased if that of........could find it convenient to comply with the request of the committee to give the matter publicity, in order that it may come to the knowledge of interested organizations and public spirited citizens of that country."

The Department will be pleased to have you present the matter of the New York meeting in the same light.

I am, gentlemen, your obedient servant,

ELIHU ROOT.

